# m^6^A target microRNAs in serum for cancer detection

**DOI:** 10.1186/s12943-021-01477-6

**Published:** 2021-12-20

**Authors:** Bo Zhang, Zhenmei Chen, Baorui Tao, Chenhe Yi, Zhifei Lin, Yitong Li, Weiqing Shao, Jing Lin, Jinhong Chen

**Affiliations:** 1 Department of General Surgery, Huashan Hospital, Fudan University, 12 Middle Wulumuqi Road, Shanghai, 200040 PR China; 2 Cancer Metastasis Institute, Fudan University, Shanghai, 200040 PR China

**Keywords:** Liquid biopsy, m^6^A, microRNA, Diagnosis, Pan-cancer

## Abstract

Recent studies have revealed the significant dysregulation of m^6^A level in peripheral blood in several cancer types and its value in diagnosis. Nonetheless, a biomarker for accurate screening of multiple cancer types has not been established based on the perspective of m^6^A modification. In this study, we aimed to develop a serum diagnostic signature based on the m^6^A target miRNAs for the mass detection of cancer. A total of 14965 serum samples with 12 cancer types were included. Based on training cohort (*n*=7299), we developed the m6A-miRNAs signature using a support vector machine algorithm for cancer detection. The m6A-miRNAs signature showed high accuracy, and its area under the curve (AUC) in the training, internal validation and external validation cohort reached 0.979 (95%CI 0.976 - 0.982), 0.976 (95%CI 0.973 - 0.979) and 0.936 (95%CI 0.922 - 0.951), respectively. In the performance of distinguishing cancer types, the m6A-miRNAs signature showed superior sensitivity in each cancer type and presented a satisfactory AUC in identifying lung cancer, gastric cancer and hepatocellular carcinoma. Additionally, the diagnostic performance of m6A-miRNAs was not interfered by the gender, age and benign disease. In short, this study revealed the value of serum circulating m^6^A miRNAs in cancer detection and provided a new direction and strategy for the development of novel biomarkers with high accuracy, low cost and less invasiveness for mass cancer screening, such as RNA modification.

**Supplementary Information:**

The online version contains supplementary material available at 10.1186/s12943-021-01477-6.

## Main text

Most newly cancer cases were usually detected in the advanced stage, which made the patients lose the best treatment opportunity and led to a poor prognosis. The early diagnosis of cancer was of great significance for reducing cancer-caused mortality, prolonging the patient survival and reducing the social burden [1]. Due to the defect of high cost, invasiveness, poor compliance especially low accuracy of existing cancer screening methods, large-scale cancer screening was neither feasible nor cost-effective based on these existed methods [2]. Considering that the early diagnosis of cancer could significantly prolong the survival of patients, a new biomarker with more effectiveness and less invasiveness for mass cancer screening was urgently needed to develop. N6-methyladenosine (m^6^A) modification, as the most common modification in mRNA, was also widely found in the mRNA, miRNA and lncRNA. The dysregulation of m^6^A modification level was closely related to tumor occurrence and progression [3, 4]. Recent studies have revealed the significant dysregulation of m^6^A level in peripheral blood in several cancer types and its value in diagnosis. Ge et al. showed that the m^6^A level in the peripheral blood of patients with gastric cancer was significantly up-regulated compared with healthy controls, and the level increased with the progression and metastasis of gastric cancer. The AUC for evaluating the diagnostic performance of m^6^A in gastric cancer was 0.929, which was significantly greater than CEA and CA19–9 [5]. Xiao et al. found m^6^A level in peripheral blood of breast cancer patients was also significantly up-regulated, and closely related to the stage, and its diagnostic value was much higher than CEA and CA153 [6]. Pei et al. revealed the level of leukocyte m^6^A was a potential non-invasive screening, monitoring and diagnostic biomarkers for non-small cell lung cancer [7]. Existing evidence showed m^6^A marker, as a key post transcriptional modification, promoted the initiation of miRNA biogenesis, such as promoting primary microRNA processing [8]. miRNA dysregulation caused by m^6^A has been confirmed to play an important role in tumor metastasis and progression [9]. The circulating miRNAs in serum had a high stability, and its expression was less affected by long-term storage at room temperature and freeze-thawing [10]. The above results suggested that development of novel diagnostic biomarkers based on m^6^A target miRNA in peripheral blood may be a potential strategy for large-scale cancer screening. In this study, we included 14,965 serum samples containing 12 cancer types, and developed the m6A-miRNAs signature based on the m^6^A target miRNAs for the mass detection of cancer. The m6A-miRNAs signature showed high accuracy, and its area under the curve (AUC) in the training, internal validation and external validation cohort reached 0.979, 0.976 and 0.936, respectively. Additionally, in the performance of distinguishing cancer types, the m6A-miRNAs signature showed superior sensitivity in each cancer type and presented a satisfactory AUC in identifying lung cancer, gastric cancer and hepatocellular carcinoma. The diagnostic performance of m6A-miRNAs was also not interfered by the gender, age and benign disease. In short, this study revealed the value of serum circulating m^6^A miRNAs in cancer detection and provided a new direction and strategy for the development of novel biomarkers with high accuracy, low cost and less invasiveness for mass cancer screening, such as RNA modification.

## Results and discussion

### Identification of candidate m^6^A target miRNAs in serum

In this study, we included 14,965 serum samples to identify the candidate m^6^A target miRNAs used for the construction of diagnostic signature. These 14,965 serum samples consisted of 12 cancer types and non-cancer controls, including gastric cancer (GC, *n* = 1417), hepatocellular carcinoma (HCC, *n* = 388), lung cancer (LC, *n* = 1573), glioma (*n* = 185), esophageal carcinoma (ESCA, *n* = 566), prostate adenocarcinoma (PRAD, *n* = 809), bladder urothelial carcinoma (BLCA, *n* = 392), ovarian cancer (OV, *n* = 338), sarcoma (SARC, *n* = 486), breast invasive carcinoma (BRCA, *n* = 1285), colorectal cancer (CRC, *n* = 242) and pancreatic adenocarcinoma (PAAD, *n* = 197) as well as the non-cancer controls (*n* = 7087). The training cohort consisted of 7299 participants with the average age 64 years (range, 1–100 years) and the female proportion 49%. The validation cohort consisted of 7298 participants with the average age 64 years (range, 3–98 years) and the female proportion 48%.

**Fig. 1:**
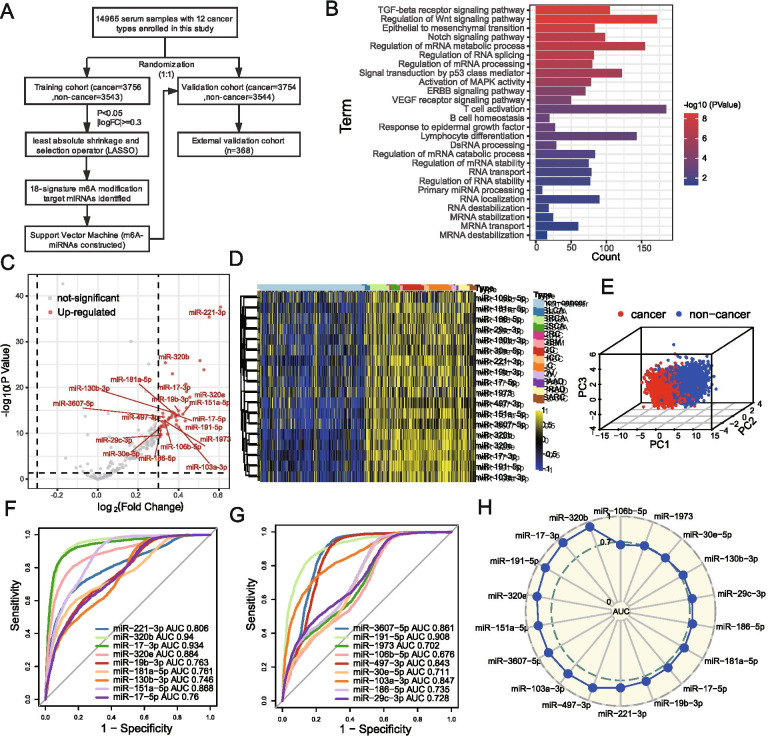
Identification of candidate m^6^A target miRNAs in serum. **A** The workflow of the establishment of serum m6A-miRNAs signature for cancer detection as well as the validation process. **B** Functional annotation for the included m^6^A target miRNAs using GO enrichment analysis. All the biological processes selected were statistically significant. The color depth of column represented *P* value and length represented enriched gene counts. **C** According to the criteria of FDR < 0.05 and |fold changes| > 1.23, the volcano plot showing the 18 candidate m^6^A target miRNAs, which were identified by LASSO, presented a higher expression level in cancer samples compared with non-cancer serum controls. Red dot, up-regulated miRNAs; Gray dot, not significant miRNAs. **D** The heatmap plotted for the expression of 18 candidate miRNAs using unsupervised hierarchical clustering in both cancer and non-cancer control groups. Yellow represented up-regulation and blue represented down-regulation. The cancer types were utilized as sample annotations. **E** Principal component analysis (PCA) for the 18 candidate m^6^A target miRNAs in cancer and non-cancer control. Two independent clusters were identified, suggesting the 18 miRNAs could well distinguished cancer samples from non-cancer controls. Red dot, cancer sample; Blue dot, non-cancer control sample. **F-G** ROC curve showing the performance of each candidate miRNA individually detecting cancer patients in the training cohort. **H** The performance of each candidate miRNA individually detecting cancer patients was validated in the internal validation cohort. The radar chart summarized the area under curve (AUC) and the AUC ranged from 0.667 to 0.94

The workflow of our study was presented in Fig. 1A. A total of 228 m^6^A target miRNAs were extracted from the ten combined serum miRNA cohort for further analyses. To explore the biological behaviors regulated by these miRNAs, we performed the GO enrichment analysis by clusterProfiler R package to reveal their potential biological pathways. As shown in Fig. 1B, these m^6^A target miRNAs were mainly enriched in some pathways involved in the cancer, immunity and RNA modification, such as the process of RNA metabolism, RNA stability, RNA localization and primary miRNA processing, as well as the signaling pathways of TGF-β receptor, VEGF receptor, WNT, and T cell activation (Table S1). Using the training cohort with 3756 cancer patients and 3543 non-cancer controls, we compared the difference of m^6^A target miRNA expression profile between the cancer and control group. miRNAs with the criterion of *p* value < 0.05 and |fold change| > 1.23 were selected for further analysis (Table S2). Finally, eighteen candidate m^6^A target miRNAs were obtained using the least absolute shrinkage and selection operator (LASSO) method to establish a serum diagnostic signature for cancer detection (Table S3). The expression of these 18 candidate miRNAs in cancer samples were significantly up-regulated compared to that in non-cancer controls (Fig. 1C). Unsupervised hierarchical clustering for the expression of these miRNAs presented a obvious separation between cancer types and controls (Fig. 1D). The principal component analysis (PCA) for the candidate m^6^A target miRNAs profiles, which was visualized by three-dimensional scatterplot, revealed two independent clusters, suggesting these 18 candidate m^6^A target miRNAs had completely different expression patterns between cancer and non-caner control groups, which laid a foundation for the construction of diagnostic signature (Fig. 1E). Subsequently, we investigated the diagnostic performance of each candidate miRNA for individually detecting cancer. The AUC of a single miRNA ranged from 0.676 to 0.940 showing by receiver operating characteristic (ROC) curve, demonstrating a certain discrimination ability of these miRNAs for cancer and non-cancer controls (Fig. 1F and G). The predictive performance of these candidate miRNAs was also well validated in the validation cohort (Fig. 1H). The above results indicated that these candidate m^6^A target miRNAs had potential as biomarkers for the detection of cancer.

### Construction of m6A-miRNAs signature for cancer detection

**Fig. 2:**
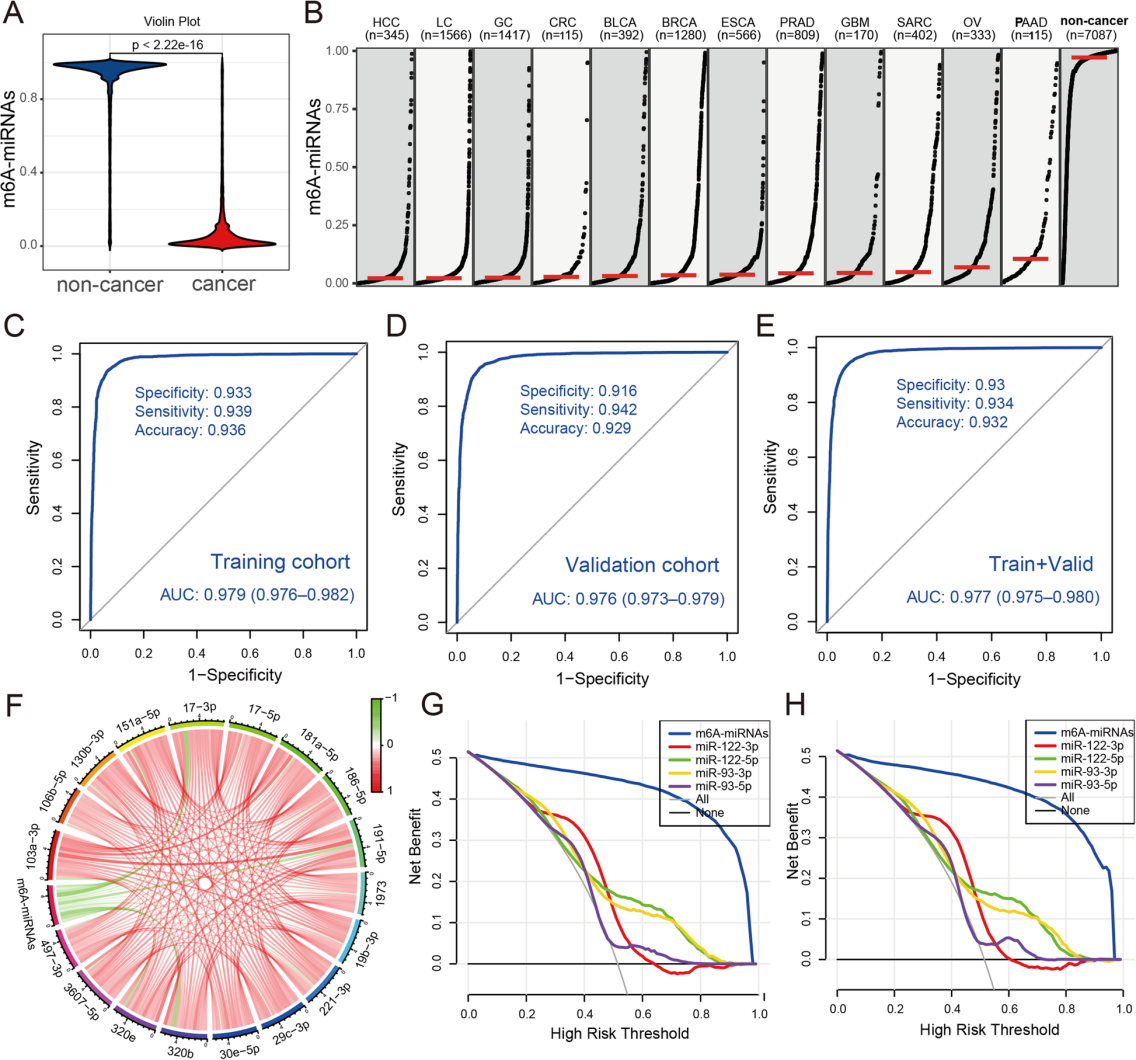
Construction of serum m6A-miRNAs signature for cancer detection. **A** Differences in the output strength of m6A-miRNAs signature between cancer and non-cancer control samples visualized by violin plot. **B** Differences in the output strength of m6A-miRNAs signature among different cancer types. The red lines represented median value of each group. **C** The ROC curve showing the diagnostic performance of m6A-miRNAs signature in distinguishing cancer from non-cancer controls in the training cohort. The area under the curve, specificity, sensitivity and accuracy were also calculated. **D** The diagnostic performance of m6A-miRNAs signature was validated in the internal validation cohort using ROC curve. **E** The diagnostic performance of m6A-miRNAs signature was investigated in the combined training and validation cohort using ROC curve. **F** Correlation between m6A-miRNAs output strength and 18 candidate m^6^A target miRNAs expression using spearman analysis. The red connection represented positive correlation and green connection represented negative correlation. The color depth of line represented the correlation coefficient. **G**, **H** In a wide range of decision threshold probability, the difference of net benefit between m6A-miRNA and other serum biomarkers using the decision curve analysis (DCA) in the training cohort **(G)** and the validation cohort **(H)**

Based on the obtained 18 candidate m^6^A target miRNAs, we used the support vector machine (SVM) algorithm to construct a diagnostic signature (named m6A-miRNAs signature) for cancer detection. The output strength of m6A-miRNAs in cancer groups was significantly lower than that in non-cancer controls (Fig. 2A). We than investigated the difference of m6A-miRNAs value between each cancer type. As shown in Table S4 and Fig. 2B, we observed HCC patients had the lowest median m6A-miRNAs (median value: 0.02211), while PAAD patients had the highest median m6A-miRNAs (median value: 0.10540), and there was a significant difference on m6A-miRNAs among HCC, LC, GC, CRC, BLCA and BRCA patients (*p* < 0.001). The m6A-miRNAs signature comprising 18 candidate miRNAs showed a high diagnostic power than each candidate miRNA alone in distinguishing cancer samples from non-cancer controls in the training cohort, with an AUC of 0.979 (95%CI, 0.976–0.982), a specificity of 93.3% (95%CI, 91.9–94.5%) and a sensitivity of 93.9% (95%CI, 92.8–95.2%). The diagnostic accuracy was 93.6% (95%CI, 93.1–94.2%) (Fig. 2C). We then applied the m6A-miRNAs signature to the internal validation cohort. Similar to the training cohort, the m6A-miRNAs signature also showed a high diagnostic performance, with the specificity of 91.6% (95%CI, 90.2–93.8%), sensitivity of 94.2% (95%CI, 92.0–95.4%) and accuracy of 92.9% (95%CI, 92.4–93.5%) (Fig. 2D). The area under the ROC curve in internal validation cohort was 0.976, with the 95%CI 0.973 to 0.979 (Fig. 2D). We also examined the m6A-miRNAs signature in the combined training and internal validation cohort. The AUC, specificity, sensitivity and accuracy were calculated and demonstrated a satisfactory diagnostic value (Fig. 2E). To further evaluate the diagnostic value of m6A-miRNAs signature, we applied the m6A-miRNAs into the external validation cohort, and a comparable area under the curve with the training cohort was obtained, with the AUC of 0.936 and 95%CI 0.922 to 0.951 (Fig. S1A). In order to explore the relationship between m6A-miRNAs and each candidate miRNA, we used the spearman correlation analysis. We observed a remarkable negative correlation between m6A-miRNAs output strength and each candidate m^6^A target miRNA expression, especially the miR-320b (coefficient: 0.557; Fig. 2F and Table S5). Previous evidence indicated the miR-320b could play a crucial role in the tumor metastasis and prognosis. Neerincx et al. showed that the expression of miR-320b was remarkably up-regulated in metastatic lesion compared to the primary colorectal cancer [11]. Jian et al. confirmed that the miR-320b level of plasma exosomes in both adenocarcinoma and squamous cell carcinoma patients was significantly overexpressed especially in squamous cell carcinoma patients compared to healthy subjects [12]. The serum level of miR-320b was also regarded as an independent biomarkers for ovarian cancer early detection [13]. Recent study demonstrated hypermethylation of miR-320b was related to the worse five-year survival in oral cancer [14]. Li et al. identified a four-miRNA prognostic signature and established a key miRNA-m^6^A related gene network based on miR-320b, which could contribute to the prognosis evaluation of patients with esophageal cancer [15]. The above results demonstrated that the m6A-miRNAs signature established based on these candidate miRNAs had a stable diagnostic performance. The subsequent calibration curve analyses presented a near perfect calibration of m6A-miRNAs in both the training and internal validation cohorts, with the predicted probability of cancer almost equal to the observed actual probability (Fig. S1B, C). The previously published studies reported the important value of miR-93 and miR-122 in pan-cancer diagnosis and prognosis [16, 17]. In the decision curve analyses, m6A-miRNAs demonstrated an absolute superiority net benefit within a wide range of decision-making threshold probabilities, compared to the miR-93 and miR-122 (Fig. 2G, H).

### Diagnostic performance of m6A-miRNAs signature in different clinical conditions and cancer types

**Fig. 3:**
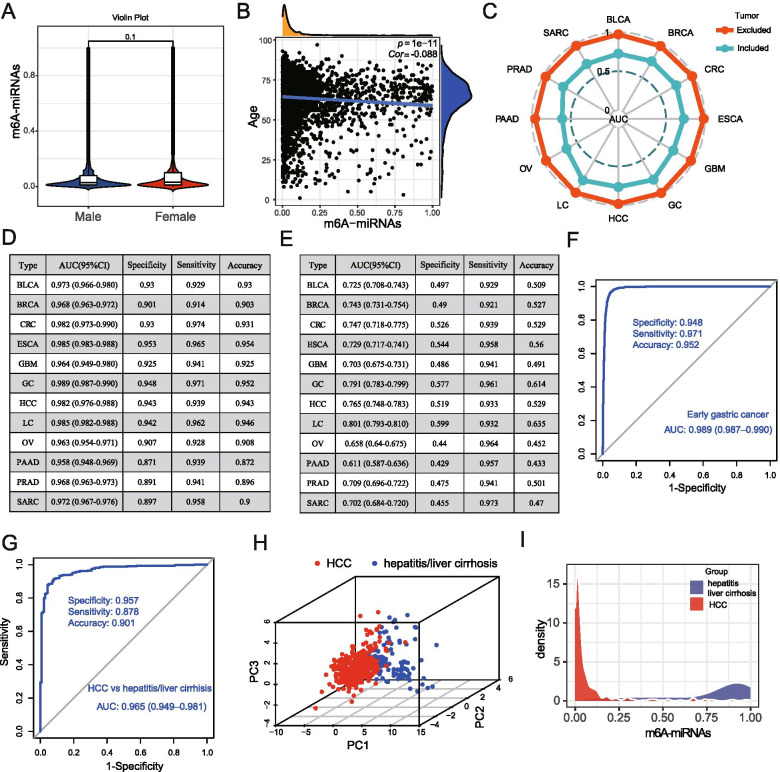
Diagnostic performance of m6A-miRNAs signature in different clinical conditions and cancer types. **A** Differences in the output strength of m6A-miRNAs signature between female and male samples visualized by violin plot. **B** Correlation between m6A-miRNAs output strength and participant age using spearman analysis. Yellow represented the density of samples at different m6A-miRNAs output strength. Blue represented the density of samples at different age. Cor, correlation coefficient. **C** The ability of m6A-miRNAs signature in distinguishing cancer types was determined by calculating the area under curve. The radar chart summarized the AUC of each cancer type. Red polyline represented the AUC value of m6A-miRNAs in distinguishing each cancer type from non-cancer controls. Blue polyline represented the AUC value of m6A-miRNAs in distinguishing each cancer type from all the mixed samples with cancer and non-cancer. **D** Summary of AUC, sensitivity, specificity and accuracy of m6A-miRNAs in distinguishing each cancer type from non-cancer controls. **E** Summary of AUC, sensitivity, specificity and accuracy of m6A-miRNAs in distinguishing each cancer type from all the mixed samples with cancer and non-cancer. **F** The ROC curve showing the diagnostic value of m6A-miRNAs signature in early gastric cancer. **G** The ROC curve showing the ability of m6A-miRNAs in distinguishing patients with hepatocellular carcinoma (HCC) and patients with hepatitis/liver cirrhosis. **H** Principal component analysis for the 18 candidate m^6^A target miRNAs in HCC and hepatitis/liver cirrhosis samples. Two independent clusters were identified. Red dot, HCC samples; Blue dot, hepatitis/liver cirrhosis samples. **I** The density of HCC samples and hepatitis/liver cirrhosis at different m6A-miRNAs output strength

Considering the inclusion of breast, ovarian and prostate cancer in our study, we tested the diagnostic performance of m6A-miRNAs signature classified by patient sex. We did not observe a significant difference on the output strength of m6A-miRNAs signature between female and male patients (*p* = 0.1, Fig. 3A). Matched with the above results, m6A-miRNAs always showed superior diagnostic performance and discrimination ability no matter in male cancers or female cancers. For male populations, the AUC was 0.987 (95%CI, 0.984–0.989), with the specificity of 94.4% (95%CI, 92.9–95.8%), sensitivity of 90.91% (95% CI: 93.4–96.4%) and diagnostic accuracy of 94.7% (95% CI: 94.2 to 95.2%) (Fig. S1D). For female populations, the AUC was 0.968 (95%CI, 0.964–0.972), with the specificity of 90.9% (95%CI, 89.2–92.5%), sensitivity of 92.7% (95% CI, 91.1–94.3%) and diagnostic accuracy of 91.8% (95% CI, 91.1 to 92.4%) (Fig. S1E). In order to reveal the influence of patient age on the diagnostic efficacy of m6A-miRNAs signature, we performed the correlation analysis and found there was no significant correlation between patient age and m6A-miRNAs output strength (cor = − 0.088, Fig. 3B). This suggested that our constructed m6A-miRNAs signature was an independent biomarker for distinguishing cancers from controls, which was not interfered by the patient’s gender and age. Then, we investigated the ability of m6A-miRNAs in distinguishing cancer types. When we combined each cancer type individually with non-cancer control samples, the m6A-miRNAs signature still showed superior ability of discrimination (Fig. 3C, red polyline). Although the ability of m6A-miRNAs signature in distinguishing each cancer type from the mixed samples of all cancer and non-cancer controls was a little weakened, the m6A-miRNAs still showed a remarkably high sensitivity (Fig. 3C, blue ployline). This meant when judging whether a patient belonged to a certain cancer type, more than 92% of patients with this cancer type could be identified by m6A-miRNAs signature, with a lower missed diagnosis rate. Here, we found the m6A-miRNAs signature for distinguishing the types of hepatocellular carcinoma, gastric cancer and lung cancer still showed a satisfactory area under the curve, with the AUC reaching 0.765, 0.791 and 0.801 respectively (Fig. 3E). In Fig. 3D and E, we summarized the diagnostic performance of m6A-miRNAs signature including AUC, specificity, sensitivity and accuracy according to cancer types. We noted that m6A-miRNAs showed a promising AUC value for the diagnosis of early gastric cancer, with a AUC of 0.989 (95%CI, 0.987–0.990), a specificity of 0.948, a sensitivity of 0.971 and accuracy of 0.952 (Fig. 3F), much higher than carcinoembryonic antigen (CEA) and carbohydrate antigen (CA19–9). Since hepatitis B and C infections were one of the main causes of HCC, and often interfered the diagnosis of HCC, we investigated the ability of m6A-miRNAs signature in distinguishing the HCC patients and patients with chronic hepatitis\liver cirrhosis. We found the diagnostic performance of m6A-miRNAs signature was not influenced by the chronic hepatitis\liver cirrhosis (AUC, 0.965; specificity, 0.957; sensitivity: 0.878; accuracy: 0.901; Fig. 3G). The diagnostic signature based on these candidate m^6^A miRNAs combination was highly accurate in distinguishing patients with HCC from the patients with chronic hepatitis\liver cirrhosis (Fig. 3H), much better than the traditional biomarker such as AFP (the performance of AFP: AUC, 0.65; specificity, 51.4%; sensitivity, 73.3%) [18]. The output strength of m6A-miRNAs signature in patients with HCC was mainly concentrated in the 0 to 0.13, hardly intersecting with the value range of patients with chronic hepatitis\liver cirrhosis (Fig. 3I). The above results indicated that the diagnostic performance of m6A-miRNAs signature may not be affected by chronic diseases.

There were several limitations in our study. Although we have demonstrated the m6A-miRNAs showed a promising AUC value for the diagnosis of early gastric cancer, considering the lack of corresponding stage information in other cancers, we could not evaluate the value of m6A-miRNAs in other cancer early diagnosis. Therefore, the performance of m6A-miRNAs signature in diagnosing other cancer with early stage was still needed to be further investigated.

## Conclusions

In conclusion, this study revealed the value of serum circulating m^6^A target miRNAs in cancer detection, and constructed a diagnostic signature m6A-miRNAs that could detect cancer with high accuracy. This signature could have the potential to become a noninvasive and cost-effective tool for large-scale cancer screening. The prospective cohort studies were needed to validate the clinical feasibility of m6A-miRNAs signature in cancer detection.

## Supplementary Information

**Additional file 1: Figure S1.** The evaluation for the diagnostic performance of m6A-miRNAs signature. **(A)** The diagnostic value of m6A-miRNAs signature was validated in the external validation cohort using ROC curve. **(B-C)** Calibration plots showing the probability of predicted vs observed cancer detected by the m6A-miRNAs signature in the training cohort **(B)**, and in the validation cohort **(C)**. **(D-E)** The ROC curve showing the diagnostic value of m6A-miRNAs signature in male populations **(D)** and female populations **(E)**. The area under the curve, specificity, sensitivity and accuracy were calculated.

**Additional file 2.**

**Additional file 3: Table S1.** Functional annotation for m^6^A target miRNAs. **Table S2.** Differential expression analysis of the m^6^A target miRNAs between cancer and non-cancer control in serum. **Table S3.** Identification of eighteen candidate m^6^A target miRNAs used for constructing diagnostic signature. **Table S4.** The median value of m6A-miRNAs signature in the 12 cancer types. **Table S5.** Spearman correlation between the m6A-miRNAs signature and 18 candidate miRNAs.

## Data Availability

All data used in this study were available in the Gene-Expression Omnibus repository (GEO; https://www.ncbi.nlm.nih.gov/geo/), and the corresponding identifier was provided in the supplementary materials.

## Abbreviations

AFP: Alpha-fetoprotein
AUC: Area under the curve
BLCA: Bladder urothelial carcinoma
BRCA: Breast invasive carcinoma
CA153: Carbohydrate antigen 153
CA19–9: Carbohydrate antigen 19–9
CEA: Carcinoembryonic antigen
CI: Confidence interval
CRC: Colorectal cancer
ESCA: Esophageal carcinoma
GC: Gastric cancer
HCC: Hepatocellular carcinoma
LASSO: Least absolute shrinkage and selection operator
LC: Lung cancer
m^6^A: N6-methyladenosine
miRNAs: microRNA
OV: Ovarian cancer
PAAD: Pancreatic adenocarcinoma
PRAD: Prostate adenocarcinoma
ROC: Receiver operating characteristic
SARC: Sarcoma
SVM: Support vector machine
TGF-β: Transforming growth factor beta
VEGF: Vascular endothelial growth factor
