# Supplementary figures and images for: m6A target microRNAs in serum for cancer detection
Source: Mol Cancer. 2021 Dec 20;20:170. doi: 10.1186/s12943-021-01477-6 (PMC8686344; doi:10.1186/s12943-021-01477-6)

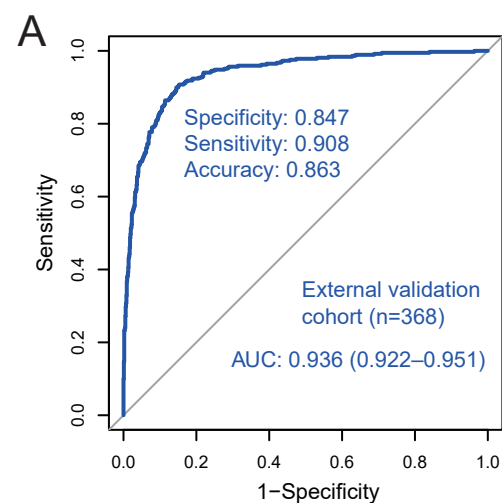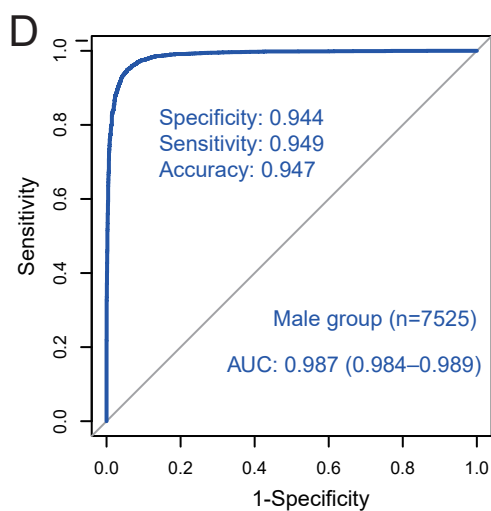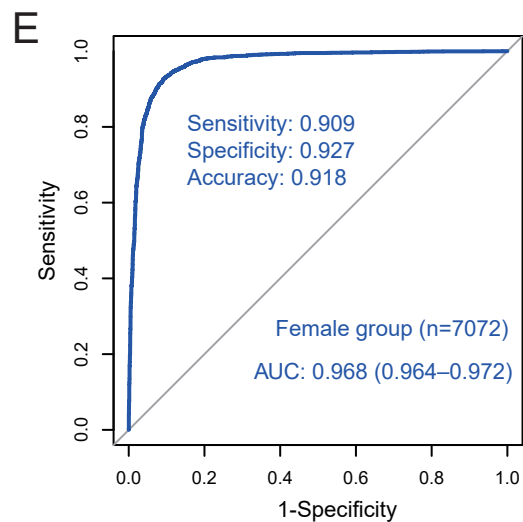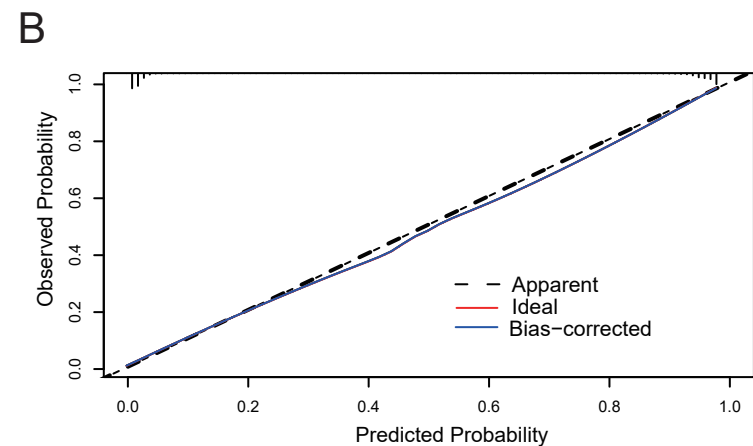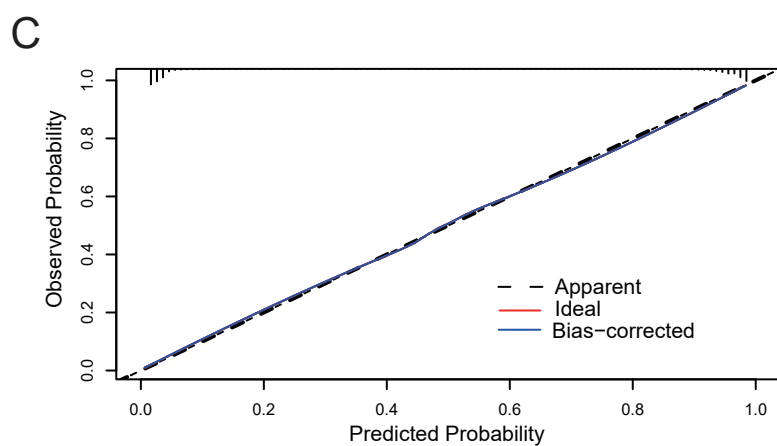

Supplement: Supplementary file 1 — Additional file 1: Figure S1. The evaluation for the diagnostic performance of m6A-miRNAs signature. (A) The diagnostic value of m6A-miRNAs signature was validated in the external validation cohort using ROC curve. (B-C) Calibration plots showing the probability of predicted vs observed cancer detected by the m6A-miRNAs signature in the training cohort (B), and in the validation cohort (C). (D-E) The ROC curve showing the diagnostic value of m6A-miRNAs signature in male populations (D) and female populations (E). The area under the curve, specificity, sensitivity and accuracy were calculated. [file 12943_2021_1477_MOESM1_ESM.pdf]
