# Supplementary material for: m6A target microRNAs in serum for cancer detection
Source: Mol Cancer. 2021 Dec 20;20:170. doi: 10.1186/s12943-021-01477-6 (PMC8686344; doi:10.1186/s12943-021-01477-6)
Supplement: Supplementary file 2 — Additional file 2. [file 12943_2021_1477_MOESM2_ESM.docx]

**m^6^A target microRNAs in serum for cancer detection**

**Supplementary materials and methods**

**Sample collection and preprocessing**

We systematically searched the public genomics data repository for the serum miRNA expression profiles and corresponding clinical information. The patients included in the case group need to be clearly diagnosed as malignant tumors by the gold standard. We excluded the patients who had received any treatment including chemotherapy, radiotherapy or surgery before serum collection. The serum samples in the control group were obtained from healthy volunteers or patients with benign diseases. Finally, a total of ten eligible serum miRNA cohorts from the Gene-Expression Omnibus (GEO) repository (GSE164174, GSE113740, GSE137140, GSE139031, GSE122497, GSE112264, GSE113486, GSE106817, GSE124158 and GSE73220) were included in this research. These miRNA cohorts were based on the 3D-Gene Human miRNA platform. After removing duplicate samples, 7087 control serum samples and 7510 cancer serum samples were gathered for further analysis. These samples consisted of 12 cancer types including gastric cancer (GC), hepatocellular carcinoma (HCC), lung cancer (LC), glioma, esophageal carcinoma (ESCA), prostate adenocarcinoma (PRAD), bladder urothelial carcinoma (BLCA), ovarian cancer (OV), sarcoma (SARC), breast invasive carcinoma (BRCA), colorectal cancer (CRC) and pancreatic adenocarcinoma (PAAD) as well as non-control controls. The normalized matrix files of each miRNA cohort were downloaded and we used the “ComBat” algorithm of sva package to perform batch correction of different serum miRNA profiles [1].

**Functional annotation of m^6^A target miRNAs**

In this study, we extracted a total of 228 m^6^A target miRNAs from the previously published studies [2-5]. In order to investigate the biological processes involved in these m^6^A target miRNAs, we performed the GO enrichment analysis using the clusterProfiler R package [6]. The FunRich 3.1.1 software was used to predict the target genes of these miRNAs [7], and then the GO functional annotation was conducted based on the predicted target gens. The biological pathway with the FDR < 0.05 were considered as significant.

**Construction of diagnostic signature for cancer detection**

In order to develop the serum diagnostic signature for distinguishing cancers from non-cancer controls, 14597 serum samples were randomly divided into training cohort and internal validation cohort at a ratio of 1:1. 3756 cancer samples and 3543 non-cancer controls were randomly assigned to the training cohort, while the internal validation cohorts consisted of 3754 cancer samples and 3544 non-cancer controls. The randomization was performed using createDataPartition function of caret R package. Moreover, except the above samples, we also collected 368 additional serum samples from each cohort as the external validation cohort. In the training cohort, we compared the difference of m^6^A target miRNAs expression between cancer samples and non-cancer controls using limma package [8]. The m^6^A target miRNAs with the p value < 0.05 and |fold change| > 1.23 was selected for further analysis. Here, we used the least absolute shrinkage and selection operator (LASSO) analyses and support vector machine (SVM) algorithm to determine the candidate m^6^A target miRNAs and establish the diagnostic signature [9, 10]. The LASSO method was implemented to shrink sparse high-dimensional data in the training cohort, and the 18 candidate m^6^A target miRNAs were finally identified for constructing a SVM model. The “kernlab” R package in R software was used to execute the SVM algorithm for classifying the binary sample (cancer vs non-cancer control) [11]. Principally, the position of all samples in the high-dimensional space was determined by SVM algorithm, of which, each axis represented a miRNA, and the specific miRNA expression of a sample defined its position on the axis. In the process of training, the SVM algorithm plotted a hyperplane that best separated the two classes according to the distance between the hyperplane and the nearest sample of each class. Different sample classes were located on each side of the hyperplane. The diagnostic index (named m6A-miRNAs signature) was generated by the predictive strength of output of SVM classifier. The R function "predict" was utilized to quantify the prediction strength on the training and validation cohorts. The m6A-miRNAs signature was established by the prediction strength of the SVM classifier output.

**Statistical analysis**

The diagnostic performance of the m6A-miRNAs signature was evaluated by the receiver operating characteristic (ROC) curve analysis, including the area under the curve (AUC), sensitivity, specificity as well as accuracy. The spearman analysis was used to calculate the correlation between the m6A-miRNAs signature output strength and candidate m^6^A miRNAs expression. The heatmap was generated with euclidean distances. The Wilcoxon test was used to conduct the difference analyses between two groups, and Kruskal-Wallis test was used for three or more groups [12]. Principal component analysis (PCA) was performed to distinguish between cancer and non-cancer groups. All data processing was done in R version 4.0.5, and statistical P value were two-side, with p< 0.05 as statistically significance. In this study, the following R packages were mainly applied: “kernlab”, “glmnet”, “caret” and “pROC” [13].

**References**

1. Johnson WE, Li C, Rabinovic A: **Adjusting batch effects in microarray expression data using empirical Bayes methods**. *Biostatistics* 2007, **8**(1):118-127.

2. Alarcon CR, Goodarzi H, Lee H, Liu X, Tavazoie S, Tavazoie SF: **HNRNPA2B1 Is a Mediator of m(6)A-Dependent Nuclear RNA Processing Events**. *Cell* 2015, **162**(6):1299-1308.

3. Berulava T, Rahmann S, Rademacher K, Klein-Hitpass L, Horsthemke B: **N6-adenosine methylation in MiRNAs**. *PLoS One* 2015, **10**(2):e0118438.

4. Ma JZ, Yang F, Zhou CC, Liu F, Yuan JH, Wang F, Wang TT, Xu QG, Zhou WP, Sun SH: **METTL14 suppresses the metastatic potential of hepatocellular carcinoma by modulating N(6) -methyladenosine-dependent primary MicroRNA processing**. *Hepatology* 2017, **65**(2):529-543.

5. Muller S, Glass M, Singh AK, Haase J, Bley N, Fuchs T, Lederer M, Dahl A, Huang H, Chen J *et al*: **IGF2BP1 promotes SRF-dependent transcription in cancer in a m6A- and miRNA-dependent manner**. *Nucleic Acids Res* 2019, **47**(1):375-390.

6. Yu G, Wang LG, Han Y, He QY: **clusterProfiler: an R package for comparing biological themes among gene clusters**. *OMICS* 2012, **16**(5):284-287.

7. Fonseka P, Pathan M, Chitti SV, Kang T, Mathivanan S: **FunRich enables enrichment analysis of OMICs datasets**. *J Mol Biol* 2021, **433**(11):166747.

8. Ritchie ME, Phipson B, Wu D, Hu Y, Law CW, Shi W, Smyth GK: **limma powers differential expression analyses for RNA-sequencing and microarray studies**. *Nucleic Acids Res* 2015, **43**(7):e47.

9. Gao J, Kwan PW, Shi D: **Sparse kernel learning with LASSO and Bayesian inference algorithm**. *Neural Netw* 2010, **23**(2):257-264.

10. Noble WS: **What is a support vector machine?** *Nat Biotechnol* 2006, **24**(12):1565-1567.

11. Karatzoglou A, Smola A, Hornik K, Zeileis A: **kernlab - An S4 Package for Kernel Methods in R**. *Journal of Statistical Software* 2004, **11**(9):1 - 20.

12. Hazra A, Gogtay N: **Biostatistics Series Module 3: Comparing Groups: Numerical Variables**. *Indian J Dermatol* 2016, **61**(3):251-260.

13. Robin X, Turck N, Hainard A, Tiberti N, Lisacek F, Sanchez JC, Muller M: **pROC: an open-source package for R and S+ to analyze and compare ROC curves**. *BMC Bioinformatics* 2011, **12**:77.
